# Role of single photon emission computed tomography (SPECT/CT) in cervical spine surgery decision making

**DOI:** 10.1007/s10143-025-03905-3

**Published:** 2025-11-07

**Authors:** Stephanie N. Serva, Elena Brindley, Angeleah Carreras, India Shelley, Santiago David Mendoza-Ayús, Steven R. Glener, Charles Intenzo, James S. Harrop, Joshua E. Heller

**Affiliations:** 1 Department of Neurological Surgery, Thomas Jefferson University Hospital, Philadelphia, PA USA; 2 Department of Radiology, Thomas Jefferson University Hospital, Philadelphia, PA USA

**Keywords:** Cervicalgia, SPECT, Computed tomography, Cervical fusion, Pain generator

## Abstract

Identifying pain generators for cervical spine patients can be challenging, particularly in those with prior surgery. Single-photon emission computed tomography (SPECT/CT) may help localize potential areas of pain generation through increased radionuclide uptake. We conducted a single-institution retrospective cohort study evaluating the concordance between MRI and SPECT/CT findings and assessing outcomes after surgery targeting spinal levels with increased uptake. Demographic, clinical, and imaging data were extracted. Degenerative changes (spondylosis, facet arthropathy, stenosis, disc disease) seen on MRI were compared with SPECT/CT to determine concordance. Primary outcomes included change in neck disability index (NDI) and visual analog scale (VAS) pain scores. A 30% reduction in NDI from baseline defined the minimal clinically important difference (MCID). Fifty-three patients with both SPECT/CT and MRI were identified. Increased radionuclide uptake was seen in 50 (94%) patients; 40 (75.5%) had concordant degenerative findings on MRI. Twenty-nine (54.7%) had prior cervical instrumentation, of whom 16 (55%) had uptake at the instrumented level, and 12 (41%) had uptake at adjacent levels. Twenty patients underwent surgery targeting SPECT-positive levels. All patients demonstrated significant improvements in NDI (mean 36.2%, *p* < 0.001) and VAS (*p* < 0.001). SPECT/CT may serve as a valuable adjunct to MRI in evaluating cervical spine pathology, particularly in complex or postoperative cases. Patients who underwent surgery directed by SPECT/CT findings experienced meaningful clinical improvements. Further prospective studies are needed to validate its utility and cost-effectiveness.

## Introduction

Cervicalgia is a common complaint, with an age-standardized prevalence rate of 27.0 per 1000 people [1]. Identifying pain generators in cervical patients is challenging, especially in those with prior surgery. There is some evidence that single photon emission computed tomography with computed tomography (SPECT/CT) can identify potential areas of pain generation as evidenced by increased radionuclide uptake [2–4]. SPECT/CT provides information about bone remodeling activity based on the detection of a radiopharmaceutical that accumulates in areas of osteoblastic activity. The increased osteoblastic activity highlights foci of mechanical stress and is overlaid with the anatomic information of a CT scan, offering higher precision than either modality could alone [5].

Conventional bone scintigraphy is a 2-D display of the entire skeletal system. SPECT is tomographic 3-D imaging of a particular anatomic region, such as the spine. In SPECT/CT, this tomographic imaging is fused with CT images of that same region, offering functional information from SPECT and anatomic information from CT. Therefore, bone SPECT/CT uniquely allows for 3-D evaluation of foci of increased bone turnover as in normal bone scintigraphy, but with anatomic colocalization on a non-contrast CT [6]. Bone turnover can therefore be more specifically localized to, for example, a single disc space versus a single facet joint, or localized in relation to specific hardware components, rather than just a generic region of the spine as in normal bone scintigraphy. This localization plays a key role in management decisions for patients with and without hardware [7].

Conventional spine imaging modalities (radiograph, CT, MRI), while incredibly valuable and standard of care, are limited to morphologic information, and patients with chronic pain, multifocal degenerative disease, and complex surgical histories pose diagnostic challenges that are often not answered by morphologic degenerative changes alone. For example, patients with severe multilevel degenerative disease on CT and MRI may have one specific level and location of increased bone turnover on nuclear medicine (NM) Bone SPECT/CT that may or may not appear as the primary focus of degenerative changes morphologically on plain CT or MRI [7]. This suggests that the area of active bone turnover may be the most active contributor to current pain as bone turnover can incite a local inflammatory reaction [8]. In combination with the baseline morphologic and stability information offered by conventional imaging, this enables more targeted intervention planning with a focus on or at least an inclusion of the level of bone turnover that may otherwise have been excluded from surgical planning based solely on conventional imaging [7].

Accurate identification of the pain generator may not only improve outcomes after cervical spine surgery but also guide targeted surgical or nonsurgical approaches. In this study, we aim to evaluate the concordance of MRI with SPECT/CT and assess patient pain outcomes when surgery was performed on spinal levels and anatomical areas with evidence of increased radionuclide uptake on SPECT/CT.

## Design and methods

### Study design

We conducted a single institution retrospective cohort study to evaluate the concordance of MRI and SPECT/CT findings and assess patient outcomes when surgery was performed on spinal levels and anatomical areas with evidence of increased radionuclide uptake on SPECT/CT. The study reviewed clinical, demographic, imaging, and outcome data from patients who underwent SPECT/CT and MRI scans between 2021 and 2024. Demographic and clinical information was recorded, including age, sex, level of spinal pathology determined by clinical and imaging documentation, dates of imaging including SPECT/CT and MRI, and dates and types of surgical spine procedures. SPECT/CT and MRI images had all been interpreted by a board certified neuroradiologist, and findings were analyzed by a trained neurosurgeon to identify concordance between reports of degeneration at each cervical level. Each patient either underwent surgical or conservative management (physical therapy, injections, medication). Primary outcome measures were assessed over an average follow-up period of 8 weeks in both the surgical and conservatively managed patient groups. The outcome measures included improvement in visual analog pain scale (VAS) and neck disability index (NDI) which were identified from clinic notes. The primary aim was to assess the concordance between SPECT/CT findings and clinical outcomes, particularly with regard to improvements in pain and disability scores. Informed consent was waived due to the retrospective nature of the study, and patient data were anonymized to ensure confidentiality. Approval for this study was provided by Thomas Jefferson University Office of Human Research Protection (IRB).

### Participants

Patients were included if they had clinical evidence of cervical pathology, were over the age of 18, and had available imaging data (SPECT/CT and MRI). We included all patients with a primary complaint of cervicalgia who had complete imaging data in the study time frame. Exclusion criteria included patients with incomplete clinical data, those who did not have both SPECT/CT and MRI imaging, and those with cervical pain secondary to trauma or infection.

### Imaging protocol

All patients underwent cervical MRI and SPECT/CT imaging within a 6 month period as part of their clinical workup.

Standard Institution NM Bone SPECT/CT Protocol: Our NM Bone SPECT/CTs use technetium 99 labelled methyl diphosphonate (Tc-99 m-MDP). MDP primarily binds to hydroxyapatite matrix of bone when exposed and binding is proportional to vascularity and osteoblast activity. Following the intravenous administration of approximately 25 mCi of Tc-99 m-MDP (methylene diphosphonate) and a delay of approximately 2–3 h, SPECT/CT imaging of the spine region(s) of interest is performed using a noncontrast, nondiagnostic CT for localization purposes on the Symbia Intevo 2-headed SPECT/CT camera (Siemens). The images are acquired in utilizing a 128 × 128 matrix, 25–30 s per view, usually 48 views per detector or head. Image processing is done via filtered back projection. The fused images are then displayed and reviewed in 3 orthogonal views (axial, coronal, sagittal). In addition, a “flash 3D” rotating image of the area of interest is displayed. CT parameters: 16 slice, 3 mm slices.

Minimum Standard Institution Spine MR protocol for degenerative disease (noting some machine to machine variation): T2 3-plane localizers, Sagittal T1 FSE, Sagittal T2 frFSE, Sagittal FSE STIR, axial T1, axial T2.

Independent radiology trainees and attendings with subspecialty training in nuclear medicine evaluate NM bone SPECT/CT for foci of increased uptake relative to background uptake on both planar and fusion CT images. Foci of increased uptake are graded as mild, moderate, and severe, and only moderate and severe foci are considered possible pain generators. Mild uptake was considered barely perceptible over background osseous uptake. Moderate uptake was perceptible as a distinct focus over background uptake on multiple planes but did not reach the highest levels measurable by the SPECT/CT heat map. Severe uptake was identified as a distinct focus of uptake with some foci of the highest measurable heatmap data. Based on a combination of findings including the quality of the scan and amount of background uptake, the level of uptake intensity, the circumscribed/focal nature of uptake, and anatomic location, these are further labelled and reported as either definite, probable or possible pain generators. Cervical MRI was performed in all patients with standard protocols for detecting degenerative changes including spondylosis, facet hypertrophy, uncovertebral arthropathy, and degenerative disc disease. The presence of degenerative changes on MRI was compared with SPECT/CT findings to determine concordance between the imaging modalities. Patients who had increased uptake on SPECT/CT and abnormal findings at the corresponding level and anatomical structure on MRI were considered concordant. Findings were localized to spinal levels and specific anatomical structures such as facet joint, uncovertebral joint, disc. Radiologists were not blinded to clinical data when providing clinically relevant reports. Inter-rater reliability was not assessed. Patient exams interpreted by standard practices of 3 attending Nuclear Medicine physicians were included.

### Data collection

Demographic data (age, sex, prior surgeries), clinical characteristics (pain history), and imaging findings were extracted from patient records. The study also recorded the presence of pre-existing cervical instrumentation and the level(s) of instrumentation. Each patient either underwent surgical or conservative management. Primary outcome measures (VAS and NDI) were assessed over an average follow-up period of 8 weeks in both the surgical and conservatively managed patient groups. A 30% reduction in NDI from the baseline represented the minimal clinically important difference (MCID) [9].

### Statistical analysis

Descriptive statistics were used to summarize patient demographics and clinical characteristics. For outcomes data, Shapiro-Wilk test was used to test for normality of data distribution. Paired t-tests were used to compare initial and follow up scores for both the VAS and NDI. A p-value of < 0.05 was considered statistically significant. The correlation between SPECT/CT findings, MRI findings, and clinical outcomes was also assessed.

## Results

A total of 53 patients with cervical spinal pathology who underwent both SPECT/CT and MRI within 6 months of each other between 2021 and 2024 were identified. The average age was 62.1 years and 27 patients (50.9%) were male. The cohort included a mix of patients with degenerative cervical conditions, some of whom had prior spinal surgery. Among the 53 patients, 50 (94%) demonstrated foci of increased uptake graded as moderate or severe, suggesting potential areas of pain generation. Of these, 40 patients (75.5%) had corresponding evidence of degenerative changes (spondylosis, facet hypertrophy, uncovertebral arthropathy, and degenerative disc disease) at the same level and anatomical structure on MRI, reflecting concordant findings between the two imaging modalities. Patient demographic data and imaging findings are listed in Table 1. An example of concordance is a patient with SPECT/CT finding of markedly increased uptake at C3-4 facet joints and MRI finding of uncovertebral hypertrophy and facet arthropathy resulting in severe foraminal stenosis at the same level. An example of discordance is a patient with SPECT/CT finding of markedly increased activity involving the left anterior C2-C3 vertebral bodies with only mild stenosis from a central disc herniation at the same level.

Twenty-nine patients (54.7%) had pre-existing cervical instrumentation, and of these, 16 patients (55%) exhibited increased uptake at the level of the instrumentation, raising concern for possible pseudarthrosis. In contrast, 12 patients (41%) had areas of increased uptake identified at adjacent spinal levels, highlighting the potential for pathology beyond the instrumented segments such as proximal or distal junctional failure. Uptake occurred at anatomic structures at the level or adjacent to the level of existing surgical cervical hardware from prior posterior or anterior fusion. For example, a patient with prior C7-T1 ACDF had increased uptake at bilateral C7-T1 facet joints and increased uptake noted about the C7-T1 hardware.

**Table 1:** Patient demographics and imaging findings

Characteristic	Value
Mean Age	62.1
Sex (Male)	27 (50.9%)
MRI/SPECT Concordance	40 (75.5%)
Prior Surgery	29 (54.7%)
Uptake on SPECT	
Level of instrumentation	16 (55%)
Adjacent level	12 (41%)

A subgroup of 20 patients underwent surgical intervention targeting the areas of increased uptake identified by SPECT/CT. The types of surgical procedures performed in the operative cohort is listed in Table 2. These patients had previously trialed and failed conservative measures including medication, physical therapy, and epidural steroid injections. Patient outcomes were assessed at 8 weeks following surgery.

**Table 2:** Surgical procedures performed in operative cohort

Surgery Type (*n* = 20)	
ACDF	
Single level	5
Multi-level	0
PCDF	
Single level	3
Multi-level	3
Foraminotomy	1
Revision	
Anterior	2
Posterior	5
Anterior and Posterior	1 (Revision ACDF + new PCDF)

At the 8 week post-operative outpatient visit, all patients reported significant improvements in the NDI with a mean improvement of 36.2% (*p* < 0.001) indicating clinically relevant improvements in function and quality of life (exceeds MCID). Patients also experienced a statistically significant improvement in VAS (*p* < 0.001) (Table 3).

**Table 3:** Surgical group outcomes

Outcome Measure	Initial Mean	Follow-Up Mean	*p* value
NDI	30.55	19.5	< 0.001
VAS	7.05	2.85	< 0.001

The remaining patients were treated conservatively. Treatment modalities included physical therapy, injections, and medication. Clinical outcomes were monitored over the same follow-up period; however, these patients did not demonstrate statistically significant improvements in either NDI (*p* = 0.526) or VAS (*p* = 0.923) (Table 4).

**Table 4:** Conservative treatment group outcomes

Outcome Measure	Initial Mean	Follow-Up Mean	*p* value
NDI	20.45	19.55	0.526
VAS	6.06	6.0	0.923

## Discussion

Identifying the optimal patient group for any surgical procedure can be challenging, especially those aimed at treating pain. This challenge is particularly evident for patients with axial neck pain, as identification of the exact pain source can be difficult. The use of SPECT/CT has allowed for more precise localization of potential pain generators at areas with increased osteoblastic activity. While a patient’s physical exam and history may point to a specific site of pain generation, traditional imaging such as MRI and CT may fail to identify the exact location in the absence of stenosis or instability emphasizing the need for additional imaging to better implicate these pain origins [10–12]. While SPECT/CT alone did not alter surgical decision making in the operative cohort, our study highlights the utility of SPECT/CT as a diagnostic adjunct to current imaging modalities in identifying the primary pain generator in patients with cervical spinal pathology. Its utility may be particularly useful in cases where conventional imaging yielded inconclusive results [3]. The presence of positive findings on SPECT/CT in patients with otherwise unremarkable MRI studies may suggest that SPECT/CT could be more sensitive to detecting subtle osseous pathology such as active facet arthropathy, early degenerative changes, or pseudarthrosis—all of which may elude detection on static anatomical imaging. Conversely, patients with MRI-positive but SPECT-negative findings may reflect chronic or static degenerative changes that are not contributing to active pain.

Compared to the literature, our study showed a similar concordance rate (75.5%) between MRI and SPECT/CT findings: Russo et al. and Lusins et al. reported a concordance rate of 70.6% and 77% respectively [13, 14]. This has been further replicated in a study of juvenile spondylosis, demonstrating a concordance rate of 78.6% [15]. Our study also correlates concordance with clinical outcomes following surgery. In our operative subgroup, all 20 patients experienced a statistically significant improvement in both NDI and VAS. These outcomes support prior findings from Tender et al. In their study, 25 patients with SPECT-positive findings who had received anterior or posterior fusion procedures were identified. After surgery, the average VAS score for neck pain decreased from 8.8 ± 1.8 to 3.92 ± 2.2 at six months (*p* = 0.019), with no new or increased pain reported [10]. Additionally, Ravindra et al. and Russo et al. both reported statistically significant improvements in patients VAS and NDI scores after surgery at sites with SPECT positive findings [13, 16]. Further, among patients with previous cervical instrumentation, SPECT/CT remained effective in localizing possible pain generators, including in cases of suspected pseudarthrosis. This is consistent with reports by Shapovalov et al. and Easton et al., who demonstrated high specificity (91–94%) and moderate sensitivity (67–79%) of SPECT/CT for detecting pseudarthrosis [17, 18].

Some reports have recommended using SPECT/CT as a replacement to traditional imaging however, Lehman et al. found that changes on MRI did not always correlate with SPECT/CT activity thus encouraging supplementation with SPECT/CT rather than complete substitution [19]. From a safety standpoint, Technetium 99 is the most widely used radionuclide to label and thus image varied markers (including the phosphates commonly used in bone imaging) because of its safety profile and favorable energy profile for imaging [20]. With a half-life of 6 h and broad compatibility with molecular markers, its use in SPECT/CT imaging is safe, well-tolerated, and poses minimal risk to patients [21].

In summary, our study reinforces the value of SPECT/CT as a complementary imaging modality in evaluating axial neck pain, especially in the setting of inconclusive MRI. Future studies should continue to refine the indications for SPECT/CT and explore its predictive value in long-term clinical outcomes.

## Limitations

This study has several limitations that should be acknowledged. The retrospective nature of this study introduces inherent biases, particularly in the selection of patients and the data collected. Prospective studies would help to mitigate these biases and provide a more robust understanding of the utility of SPECT/CT in guiding treatment decisions. The sample size of 53 patients is relatively small, which limits the statistical power of the study. While the results suggest a promising role for SPECT/CT in identifying pain generators and guiding surgical decisions, larger studies with more diverse patient populations are needed to confirm the findings and improve generalizability. The follow-up period for patients in this study was limited, which restricts our ability to assess the long-term effectiveness of surgery guided by SPECT/CT findings. Longer follow-up would be necessary to determine the durability of improvements in pain and disability, and to assess potential long-term complications or the recurrence of pain. The study did not account for all potential confounding variables that could influence treatment outcomes, including the type of surgery performed, the presence of comorbidities, and the level of cervical instrumentation. These factors may have contributed to variability in outcomes, particularly in terms of VAS and NDI improvement. Further studies with more detailed control of these variables with comparison to a conservative management cohort would help clarify their impact on treatment efficacy. Further, a prospective design would reduce bias and enable more standardized data acquisition.

## Conclusion

Our study underscores the clinical utility of SPECT/CT as a complementary imaging modality in the evaluation and management of cervical spine pathology, particularly in patients with axial neck pain and inconclusive MRI findings. By identifying regions of increased radionuclide uptake—often reflecting active osseous pathology such as facet arthropathy or pseudarthrosis—SPECT/CT offers added diagnostic value that can enhance clinical decision-making. The ability of SPECT/CT to pinpoint areas of increased radionuclide uptake, indicative of heightened osteoblastic activity, provides critical insights into the underlying causes of pain in patients, including those with prior cervical spine surgery.

The observed concordance rate of 75.5% between MRI and SPECT/CT findings is consistent with prior studies, yet our analysis further demonstrates that patients with SPECT/CT-guided surgical targeting experienced statistically significant improvements in NDI and VAS, These findings suggest that incorporating SPECT/CT into preoperative assessment may improve surgical precision and patient outcomes, particularly in complex cases such as those with prior instrumentation or adjacent segment disease.

Importantly, our results emphasize that SPECT/CT should not replace conventional imaging, but rather serve as a diagnostic adjunct, particularly when MRI findings are equivocal or discordant with clinical presentation. Despite the promising findings, the retrospective design, small sample size, and short follow-up period present limitations to the study. Future prospective, multi-center studies with larger patient cohorts and longer follow-up are necessary to validate the role of SPECT/CT in cervical spine surgery and to address potential confounding variables. Investigating the cost-effectiveness of incorporating SPECT/CT into routine spine care and evaluating its predictive value for long-term outcomes would further clarify its role in treatment algorithms for cervical spine pain.

## Data Availability

No datasets were generated or analysed during the current study.
